# EEG Characteristics and Diagnostic Implications in Childhood Headache: A Multi-Center Study

**DOI:** 10.3389/fneur.2020.569486

**Published:** 2020-10-08

**Authors:** Young Il Rho, So Hyun Kim, Hoon-Chul Kang, Yun-Jin Lee, Young Ok Kim, Sung Koo Kim

**Affiliations:** ^1^Department of Pediatrics, College of Medicine, Chosun University, Gwangju, South Korea; ^2^Department of Pediatrics, College of Medicine, Hallym University, Chuncheon, South Korea; ^3^Department of Pediatrics, College of Medicine, Yonsei University, Seoul, South Korea; ^4^Department of Pediatrics, College of Medicine, Pusan University, Busan, South Korea; ^5^Department of Pediatrics, College of Medicine Chonnam National University, Gwangju, South Korea

**Keywords:** headache, migraine, electroencephalogram (EEG), epileptiform discharge, preventive medication, childhood

## Abstract

**Introduction:** Epilepsy and migraines are frequently observed as comorbidities, with the occurrence of one disorder increasing the probability of the other. The aim of our study was to evaluate the EEG characteristics by the type of headache and the implications of EEGs in headache patients, comparing the clinical characteristics and treatments between the headache patients with normal and abnormal EEGs.

**Methods:** We conducted a retrospective analysis reviewing the medical records of 259 patients with headaches who visited the pediatrics departments of five university hospitals and underwent EEGs over a period of 3 years. Based on the data entered, analyses of the following items were conducted: (1) comparison of the EEG abnormalities by the type of headache and the characteristics of the EEG findings and (2) comparison of the clinical characteristics between patients with normal and abnormal EEGs.

**Results:** Of the 259 patients, 31 showed abnormal EEGs, while 228 had normal EEGs. Of the 31 patients with abnormal EEGs, 17 showed epileptiform discharges, and 11 showed rhythmic slowing. The frequency of EEG abnormalities was significantly high in patients with migraines with auras than other types of headache. The Pediatric Migraine Disability Assessment (PedMIDAS) score was significantly higher in the abnormal EEG group compared with the normal EEG group (*p* = 0.001).

**Conclusion:** The results of this study suggest that the abnormal EEG group had more significant disruptions in their daily lives due to headaches than the normal EEG group and that patients with migraines with aura may need EEGs and they might also have overlapping pathophysiologic mechanisms with epilepsy.

## Highlight

- Of the 259 patients, 31 (12%) showed abnormal EEGs, and 228 (88%) had normal findings.
- Of the 31 patients with abnormal EEGs, 17 showed epileptiform discharges (9 had focal spikes and 8 had generalized spikes), and 11 showed rhythmic slowing (7 showed focal slowing, and 4 showed generalized slowing).
- Migraines with aura showed more EEG abnormalities than other types of headaches. In the patients with headaches with epileptiform discharges, the PedMIDAS scores were higher, and more anticonvulsants were prescribed prophylactically than those of the normal group. These findings imply that patients with migraines with aura may need EEGs, and they might also have overlapping pathophysiologic mechanisms with epilepsy, which can distinguish them from other types of headaches.

## Introduction

A primary headache is diagnosed through medical history and physical examination. If a specific cause, such as a brain tumor or epilepsy, is suspected, the patient undergoes brain imaging or EEG testing to differentiate it from a secondary headache (1). Epilepsy and migraines are frequently observed as comorbidities, with the occurrence of one disorder increasing the probability of the other (2–5). Migraine occurs in about one-fourth of the patients with epilepsy, whereas epilepsy is present in 8–15% of the patients with migraines (6). An EEG is a non-invasive test and is useful for studies of pathophysiology in migraine patients. For these reasons, it is often prescribed as a first-line evaluation in migraine patients. However, the European Federation of Neurological Societies (EFNS) guidelines for the diagnosis of non-acute headaches report that an interictal EEG is not routinely indicated for headache diagnosis (2). The usual indication of an EEG in headache patients is for a differential diagnosis when a serious doubt of epileptic seizure exists. This kind of situation may especially emerge in headache patients with atypical auras or episodic loss of consciousness (7). If a patient has a headache with a visual aura or brainstem aura, an EEG can be performed to differentiate its symptoms from those of epilepsy. Piccinelli et al. reported on electroencephalogram abnormalities in 12.8% of all children with headaches (8) and more commonly in children manifesting migraines with aura (9). Study of electroencephalogram variations in pediatric migraines and tension-type headaches indicate that electroencephalogram abnormalities are particularly prevalent in migraines, especially during headache attacks (10).

The aim of our study was to evaluate the frequency of EEG abnormalities in patients with headaches, the EEG characteristics by the type of headache, and the implications of EEGs in headache patients, comparing the clinical characteristics and treatments between headache patients with normal and abnormal EEGs.

## Methods and Materials

We conducted a retrospective analysis by reviewing the medical records of 259 patients with headaches who visited the department of pediatrics in five university hospitals and underwent EEGs over a period of 3 years. Electroencephalography was performed when the medical history or physical examination of the patients showed symptoms of a suspected seizure, such as visual or brainstem auras, the patient's lack of response to drug treatment, or continued headaches. Patients with a past medical history of unprovoked seizures, epilepsy, mental retardation, or significant abnormal brain imaging—except incidental benign lesions, such as small pineal cyst, arachnoid cyst, or venous anomaly—were excluded from the study. We retrospectively reviewed the medical records and collected information on age, sex, headache type, headache frequency, severity, duration, EEG and neuroimaging results, and preventive medications. The headache questionnaire form in the medical record included a family history of headache and characteristics of the headaches, such as frequency, duration, location, signs, and accompanying symptoms, severity, and disability caused by the headaches. The headaches were classified according to the International Classification of Headache Disorders (ICHD) criteria. Primary headaches were classified as migraines without aura, migraines with aura, probable migraine, tension headaches, probable tension-type headaches, and other headaches. The severity of headache was assessed using a visual analog scale (VAS: 0–10; 0 = no pain, 10 = most severe pain). Disability in daily life from headaches was assessed using Pediatric Migraine Disability Assessment (PedMIDAS) scores. The scores reflect the number of days that school, home, social, and recreational activities have been hampered by headaches over the past 3 months. We evaluated the following items based on the data of the headache patients who underwent EEGs: (1) the frequency of EEG abnormalities in patients with headaches, (2) comparison of the EEG abnormalities by the type of headache and the characteristics of the EEG findings, (3) comparison of the clinical characteristics and treatment between the patients with normal and abnormal EEGs, and (4) the diagnostic value of EEGs for patients with headaches.

Statistical analyses were performed using SPSS version 22 (IBM, Armonk, NY, USA). A chi-square test was used to compare the frequency of the two groups and mean comparisons were performed by *t*-tests for normal distributions and by the Mann-Whitney *U*-test for non-normally distributed data. The relationship between EEG abnormality and PedMIDAS score or headache type was analyzed by logistic regression to adjust for age, sex, and headache types.

In all cases, statistical significance was indicated by *p* < 0.05. This study was approved by the Institutional Review Board of Hallym University Dongtan Sacred Heart Hospital. Informed consent was waived due to the retrospective nature of the study.

## Results

**Figure 1:**
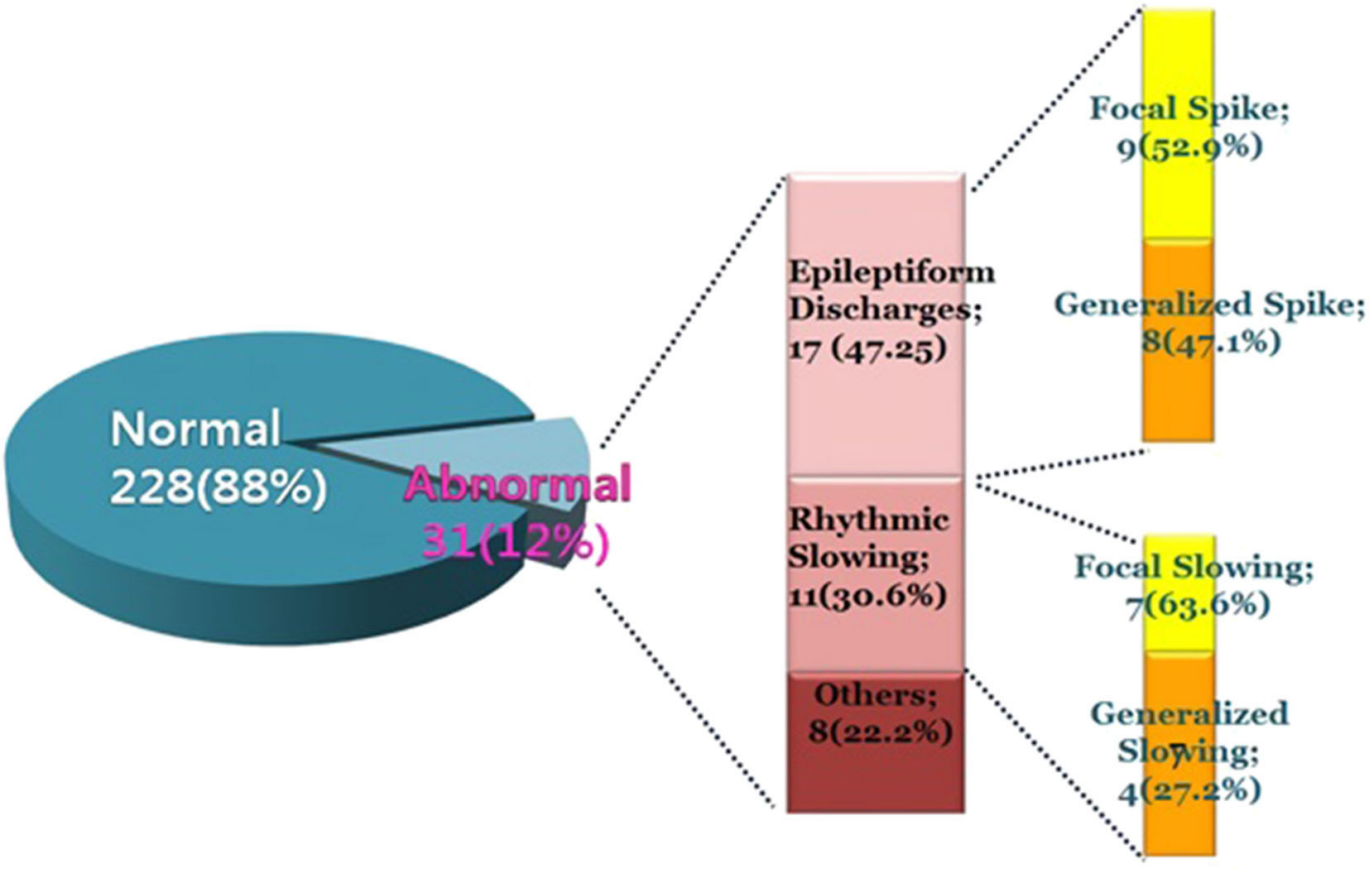
Classification of abnormal electroencephalogram (EEG) findings.

**Figure 2:**
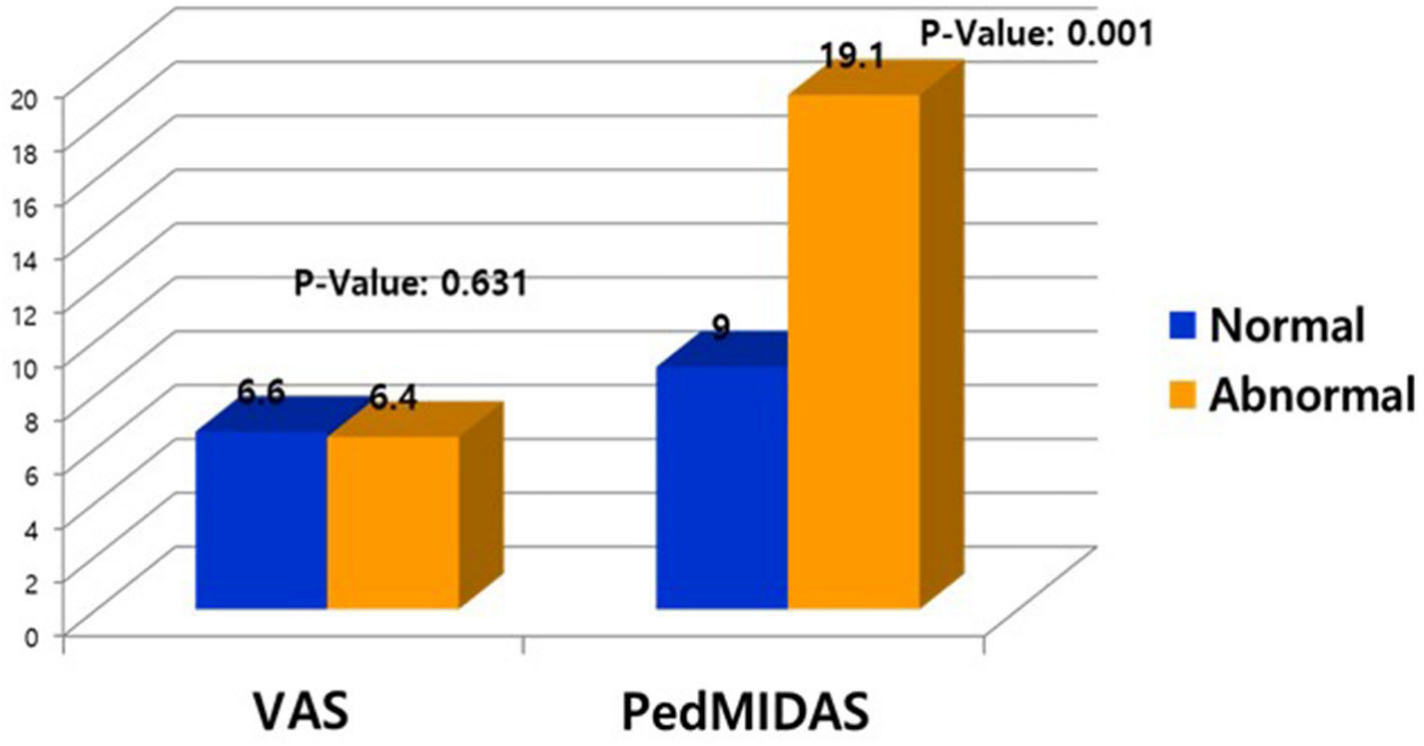
Comparison of visual assessment score (VAS) and Pediatric Migraine Disability Assessment score (PedMIDAS) between patients with normal and abnormal electroencephalograms (EEG).

**Figure 3:**
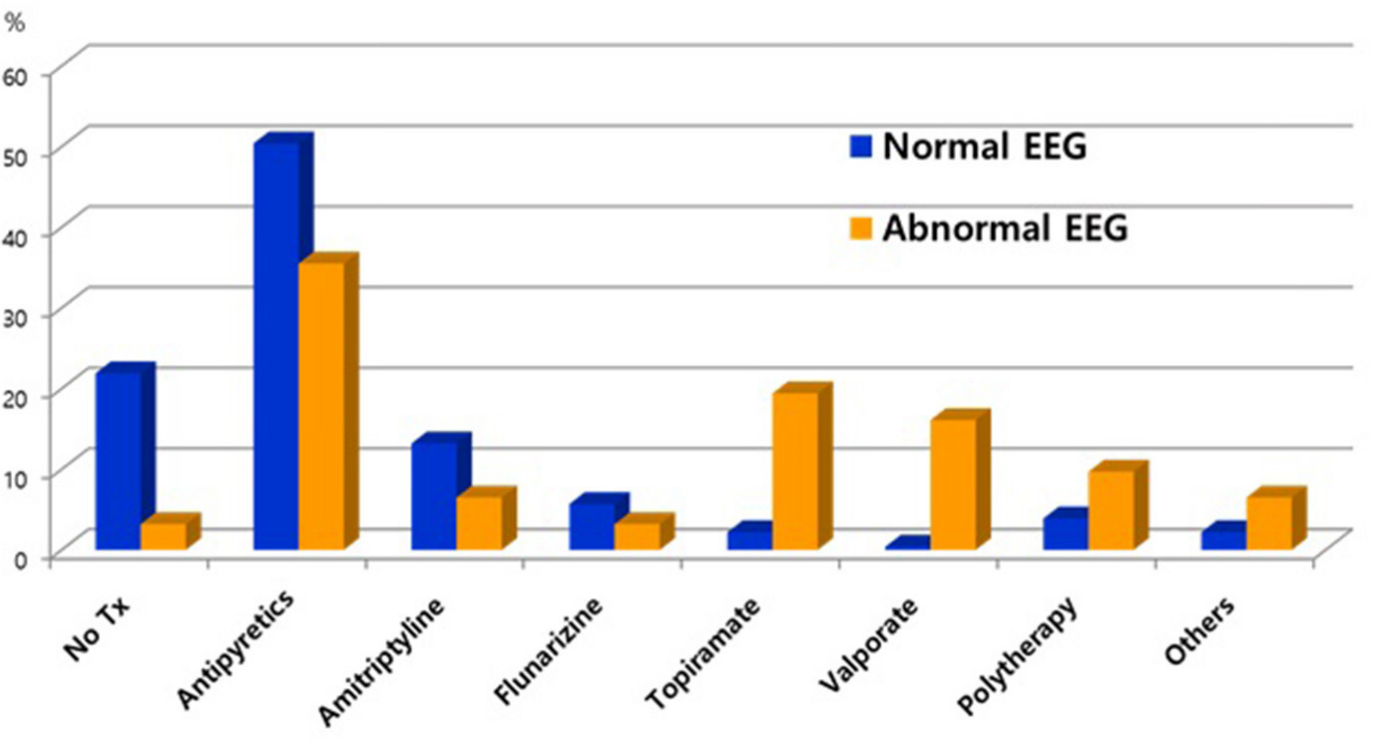
Comparison of preventive therapy between patients with normal electroencephalograms (EEG) and epileptiform discharges.

In 259 patients with headaches who underwent EEGs, the ratio of males to females was 1:1.03 and the mean age was 11.3 ± 3.4 years old. The most common type of headache was migraines without aura (108 patients), followed by 41 patients with migraines with aura, 45 with probable migraines, 11 with tension-type headaches, and 5 with probable tension-type headaches. EEGs were mainly performed for the migraine patients. Of the 259 patients, 31 (12%) showed abnormal EEGs and 228 (88%) had normal findings (Table 1). Of the 31 patients with abnormal EEGs, 17 showed epileptiform discharges (9 had focal spikes, and 8 had generalized spikes), and 11 showed rhythmic slowing (7 showed focal slowing, and 4 showed generalized slowing) (Figure 1). The frequency of EEG abnormalities was significantly different according to headache types. Ten (24.2%) patients with migraines with aura had abnormal EEGs, and 12 (11.1%) patients with migraines without aura had abnormal EEGs (Table 2). There were no differences in sex, age, or family history of headache or epilepsy between the patient groups with normal EEGs and abnormal EEGs. There were also no differences in headache frequency, characteristics, duration, or location (Table 3). The PedMIDAS score, which assesses the severity of headaches, was significantly higher in the abnormal EEG group than in the normal EEG group (*p* = 0.001) (Figure 2). EEG abnormalities were significantly related to PedMIDAS score and migraine with aura, respectively, when age, gender, and headache types were adjusted with logistic regression analysis (*p* = 0.007 adjusted odds ratio 1.034, 95% CI 1.009–1.060, *p* = 0.030, adjusted odds ratio 2.874, 95% CI 1.109–7.447). In a comparison of neuroimaging findings according to EEG findings, MRIs were performed in 76.3% of the normal and 80.6% of the abnormal EEG group and there was also no significant difference between the two groups (Table 4). Brain tumors were seen in one case of rhythmic focal slowing on the EEG but were excluded from statistical analysis. Among the 17 headache patients with epileptiform discharges, 8 patients had migraines with aura, 5 had migraines without aura, 1 had a probable migraine, 1 had migraine with aura and epilepsy, 1 had a tension-type headache, and 1 had another type of headache (Table 5). The most common preventive therapy for patients with normal EEGs was amitriptyline, followed by flunarizine. The patients with abnormal EEGs were treated most commonly with antiepileptic drugs such as topiramate and valproate (Figure 3).

**Table 1:** Clinical characteristics of the headache patients with electroencephalograms (EEG).

**Headache patient with EEG (*n =* 259)**	**Number (%)**
Sex (male/female)	127/132 (1:1.03)
Age	11.3 ± 3.4
**Headache type**	
Migraine without aura	108 (41.7)
Migraine with aura	41 (15.8)
Probable migraine	45 (17.4)
Tension-type headache	11 (4.2)
Probable tension-type headache	5 (1.9)
Others	49 (18.9)
**EEG result**	
Normal	228 (88.0)
Abnormal	31 (12.0)

**Table 2:** Electroencephalogram (EEG) findings according to headache types in the patients.

		**Migraine without aura (*n =* 108)**	**Migraine with aura (*n =* 41)**	**Probable migraine (*n =* 45)**	**Tension-type HA (*n =* 11)**	**Probable tension- type HA (*n =* 5)**	**Others (*n =* 49)**	***P*-value**
Abnormal EEG		12 (11.1)	10 (24.4)	1 (2.2)	2 (18.2)	0 (0)	6 (12.2)	0.047
Epileptiform discharge	Focal	6	2	1	0	0	0	
	General	2	2	0	2	0	2	
Slowing	Focal	3	1	0	0	0	3	
	General	3	1	0	0	0		
Others		3	3	0	0	0	2	

**Table 3:** Comparison of clinical characteristics between headache patients with normal and abnormal electroencephalograms (EEG).

		**Normal (*n =* 228) (%)**	**Abnormal (*n =* 31) (%)**	***P*-value**
Sex (M:F)		110:118	17:14	0.491
Age (years)		11.4 ± 3.4	10.5 ± 3.7	0.168
Family history of headache		82 (36)	14 (45.2)	0.320
Family history of epilepsy		3 (1.3)	0 (0.0)	0.521
Duration of illness	24 h	13 (9.4)	0 (0.0)	0.906
	1 to <7 d	33 (23.9)	3 (37.5)	
	1 wk to <1 mo	23 (16.7)	1 (12.5)	
	1 mo to <6 mo	23 (16.7)	1 (12.5)	
	6 mo to 1 yr	18 (13.0	1 (12.5)	
	>1 yr	28 (20.3)	2 (25.0)	
Frequency	<1/mo	31 (14.0)	1 (3.1)	0.745
	1–3/mo	47 (21.3)	8 (25.8)	
	1/week	7 (3.2)	2 (6.5)	
	2–3/week	37 (16.0.7)	10 (32.3)	
	4–6/week	18 (8.1)	3 (9.7)	
	Every day	81 (36.7)	7 (22.6)	
Headache characteristics	Throbbing	77 (34.5)	4 (13.8)	0.227
	Pressing	75 (33.6)	11 (37.9)	
	Squeezing	16 (7.2)	2 (6.9)	
	Stabbing	32 (14.3)	8 (27.6)	
	Pinching	1 (0.4)	0 (13.8)	
	Others	22 (9.9)	4 (12.9)	
Duration	<1 min	13 (5.9)	3 (10.3)	0.406
	2–15 min	6 (2.7)	1 (3.4)	
	16–30 min	18 (8.1)	5 (17.2)	
	31–59 min	62 (27.9)	5 (17.2)	
	1–72 h	117 (52.7)	14 (48.3)	
	>3 d	6 (2.7)	1 (3.4)	
Location	Bitemporal	57 (25.0)	7 (22.6)	0.220
	Left temporal	23 (10.1)	5 (16.1)	
	Right temporal	17 (7.5)	6 (19.4)	
	Frontal	47 (20.6)	2 (6.5)	
	Vertex	13 (5.7)	4 (12.9)	
	Occipital	19 (8.3)	2 (6.5)	
	Periorbital	3 (1.3)	0 (0.0)	
	Posterior orbital	2 (0.9)	0 (0.0)	
	Whole	30 (13.2)	3 (9.7)	
	Others	13 (5.7)	1 (3.2)	

**Table 4:** Comparison of neuroimaging findings between patients with normal and abnormal electroencephalograms (EEG).

			**Normal (*n =* 228)**	**Abnormal (*n =* 31)**	***P*-value**
Neuroimaging	Not done CT MRI		36 (15.8) 18 (7.9) 174 (76.3)	4 (12.9) 2 (6.5) 25 (80.6)	0.866
	Result (total)	Normal	162 (84.4)	19 (70.4)	0.174
		Abnormal	30 (15.6)	8 (29.6)	

*CT, computed tomography; MRI, magnetic resonance image*.

**Table 5:** Acute and preventive medication of headache patients with epileptiform discharges.

	**Medication**	**Number**	**Diagnosis**
Focal	No treatment	1	Probable migraine
	Ibuprofen	2	Migaine without aura
	Amitriptyline	1	Migraine without aura
	Flunarizine	1	Migraine with aura
	Valproate	2	Migraine with aura
	Topiramate	2	Migraine without aura
Generalized	Antibiotics	1	Others (sinusitis)
	Ibuprofen	1	Migraine with aura
	Valproate	2	Migraine with aura
	Topiramate	2	Migraine with aura
	Topiramate, oxcarbazepine	1	Migraine with aura with epilepsy
	Topiramate, levatriacetam,	1	Tension type headache
	Oxcarbazepine		

## Discussion

EEGs are not routinely recommended for headache patients because they do not help with a primary headache diagnosis. They are helpful only in patients with visual symptoms or brainstem auras. Epilepsy and headaches often occur simultaneously in the same patient. In patients with migraines, epilepsy is present in 5.9% (range 1–17%), which is high compared with the prevalence rate of 0.5–1% in the general population (3). Similarly, 14.7% of the epilepsy patients have migraines (range 8–24%), which is high compared with the prevalence rate of 12% in the general population (4–6). Generally, migraines occur in about one-fourth of the patients with epilepsy, whereas epilepsy is present in 8–15% of the patients with migraines (6). There are a few studies on the characteristics of EEGs according to the type of headache and the implication of EEGs for headache patients. EEG abnormalities were reported to vary (8.8–20%) in pediatric headache patients (8, 10, 11), and the most common abnormalities were epileptiform discharge seen in 0.4–20% of the patients (12–18). Piccinelli et al. reported electroencephalographic abnormalities in 12.8% of all children with headaches (8). In our study, of the 259 headache patients who underwent EEGs, 31 (12%) showed abnormal EEGs, consisting of 17 (6.6%) epileptiform discharges (9 with focal spikes and 8 with generalized spikes) and 11 with slowing (7 with focal slowing and 4 with generalized slowing) while 228 (88%) had normal findings.

The mechanism of the EEG abnormalities seen in headache patients, unlike the general population, may be different in migraine patients. Migraine patients have a potential for intrinsic or genetic predispositions to cortical neuron hyperexcitability. It has been suggested that the threshold of excitability of the cortical neurons causing headaches in migraine patients is lower than the threshold of cortical neuronal excitation causing seizures in epilepsy patients (19). This hypothesis explains why the prevalence of headaches in epilepsy patients was greater than the prevalence of epilepsy in headache patients and the underlying pathophysiologic mechanisms in abnormal EEGs in headache patients.

The EEG abnormalities differed by the type of headache (10) and the characteristics of the EEG findings. According to a previous study, 36% (18/50) of the migraine patients and 12% (6/50) of the tension-type headache patients revealed specific electroencephalogram abnormalities in headache attack electroencephalograms (*p* < 0.05). In electroencephalograms taken during headache-free periods, 16% (8/50) of the migraine group and 2% (1/50) of the tension-type headache group revealed abnormalities (*p* < 0.05) (17). In our study, according to headache types, there was a significantly high frequency of EEG abnormalities. Ten (24.4%) patients with migraines with aura had abnormal EEGs, and 12 (11.1%) patients with migraines without aura had abnormal EEGs (Figure 2). These findings suggest that patients with migraines with aura may need EEGs and they might also have overlapping pathophysiologic mechanisms with epilepsy, which can distinguish them from other types of headaches.

There were no differences in the demographic data and clinical characteristics of the headache patients with normal and abnormal EEGs except for the PedMIDAS scores, which assess the disability in daily life due to headaches. The scores were statistically significantly higher in the abnormal EEG group compared to the normal EEG group in our study (Figure 3). Because of this, the headache patients with abnormal EEGs had more significant disruptions in their daily lives due to headaches than the patients with normal EEGs. We may need to be concerned with focal slowing in the EEG that might be due to brain tumors, as was seen in one case of rhythmic focal slowing in the EEG in this study, and even with which cases were excluded from statistical analysis. In a small population study, headache patients with epileptiform discharge could effectively prevent headaches by taking anticonvulsants (20). In our study, headache patients with epileptiform discharges more frequently used anticonvulsants as headache prevention drugs than the patients with normal EEGs. Further prospective studies comparing the effectiveness of preventive therapy with anticonvulsants in patients with normal and epileptiform discharges in the EEGs are needed. Rare cases have been reported of pure or isolated ictal epileptic headaches occurring as the sole epileptic manifestation (21–26). Rho et al. (27) found that one-third of the patients with epilepsy and headaches had a headache first, and more than half had a history of headaches when they first visited a hospital for the evaluation of a seizure. Therefore, especially in headache patients with epileptiform discharges in the EEG, the occurrence of epilepsy should be monitored by long-term follow-up. In view of the usefulness of EEGs to differentiate epilepsy in headache patients, our study was limited because only one of the 17 patients with epileptiform discharges was finally diagnosed with epilepsy, but the follow-up period was short, and anticonvulsants were usually used as a headache prevention agent to suppress the occurrence of seizures. Prospective follow-up studies of long-term prognosis and the occurrence of epileptic convulsions in headache patients with abnormal EEGs are needed because epilepsy patients with comorbid migraines initially have a headache as the only symptom (19, 28). Although the patient was not included this study, headache was the only ictal symptom in an epileptic patient who was diagnosed incidentally when an interictal EEG was being recorded with the simultaneous onset of ictal epileptiform discharges and headaches. A limitation of this study was the retrospective nature of the chart review, which made it difficult to determine the long-term prognosis of the patients with abnormal EEGs.

## Conclusion

We identified the frequency and characteristics of abnormal EEGs according to the type of headache. Migraines with aura showed more EEG abnormalities than other types of headaches. In the patients with headaches with epileptiform discharges, the PedMIDAS scores were higher than those of the normal group, and more anticonvulsants were prescribed prophylactically.

To determine the value of EEGs in headache patients, more follow-up studies are required for headache patients with epileptiform discharges.

## Data Availability Statement

The raw data supporting the conclusions of this article will be made available by the authors, without undue reservation.

## Ethics Statement

The studies involving human participants were reviewed and approved by The Institutional Review Board of Hallym University Dongtan Sacred Heart Hospital. Written informed consent from the participants' legal guardian/next of kin was not required to participate in this study in accordance with the national legislation and the institutional requirements.

## Author Contributions

The first author and the corresponding author each contributed 30% and the remaining co-authors contributed 10% each. All authors contributed to the article and approved the submitted version.

## Conflict of Interest

The authors declare that the research was conducted in the absence of any commercial or financial relationships that could be construed as a potential conflict of interest.
